# A separate-dural-incision method of extradural dumbbell spinal schwannoma resection: cumulative experience at a single center

**DOI:** 10.1186/s12893-024-02498-w

**Published:** 2024-07-10

**Authors:** Li Jia, Minghui Zeng, Zhiyu Xi, Lin Wang, Jiang Liu

**Affiliations:** Department of Neurosurgery, Division of Life Science and Medicine, The First Affiliated Hospital of USTC (Anhui Provincial Hospital, University of Science and Technology of China, Hefei, 230036 Anhui China

**Keywords:** Spinal schwannoma, Dumbbell schwannoma, Extradural tumor, Microsurgery

## Abstract

**Objective:**

To present our experience in the surgical management of completely extradural dumbbell spinal schwannomas with a new surgical strategy.

**Method:**

This study is a case series of patients treated at the Neurosurgery Department of the First Affiliated Hospital of USTC, between January 2018 and June 2021.

**Results:**

24 patients met the inclusion criteria, with cervical and lumbar spines being the most frequent locations. All patients underwent surgical treatment. Total gross resection was accomplished in all patients. Two cases had numbness and no case exhibited motor deficit. There was no postoperative CSF leakage or wound infection.

**Conclusion:**

Based on a limited number of observations, we conclude that our technique was feasible and effective for the treatment of extradural dumbbell spinal schwannomas.

**Clinical trial:**

http://www.chictr.org.cn/, No. ChiCTR2400086171.

## Introduction

Dumbbell tumors, referring to separate tumors that connect and have two or more separate regions such as intradural space, epidural space, and locations outside the paravertebral space [1]. About 20% of primary spinal cord tumors were dumbbell tumors, [1, 2] and schwannomas account for 70% of Spinal dumbbell tumors [1]. Total resection of spinal schwannomas is generally the ideal goal because it reduces the chance of recurrence [3, 4].

**Fig. 1:**
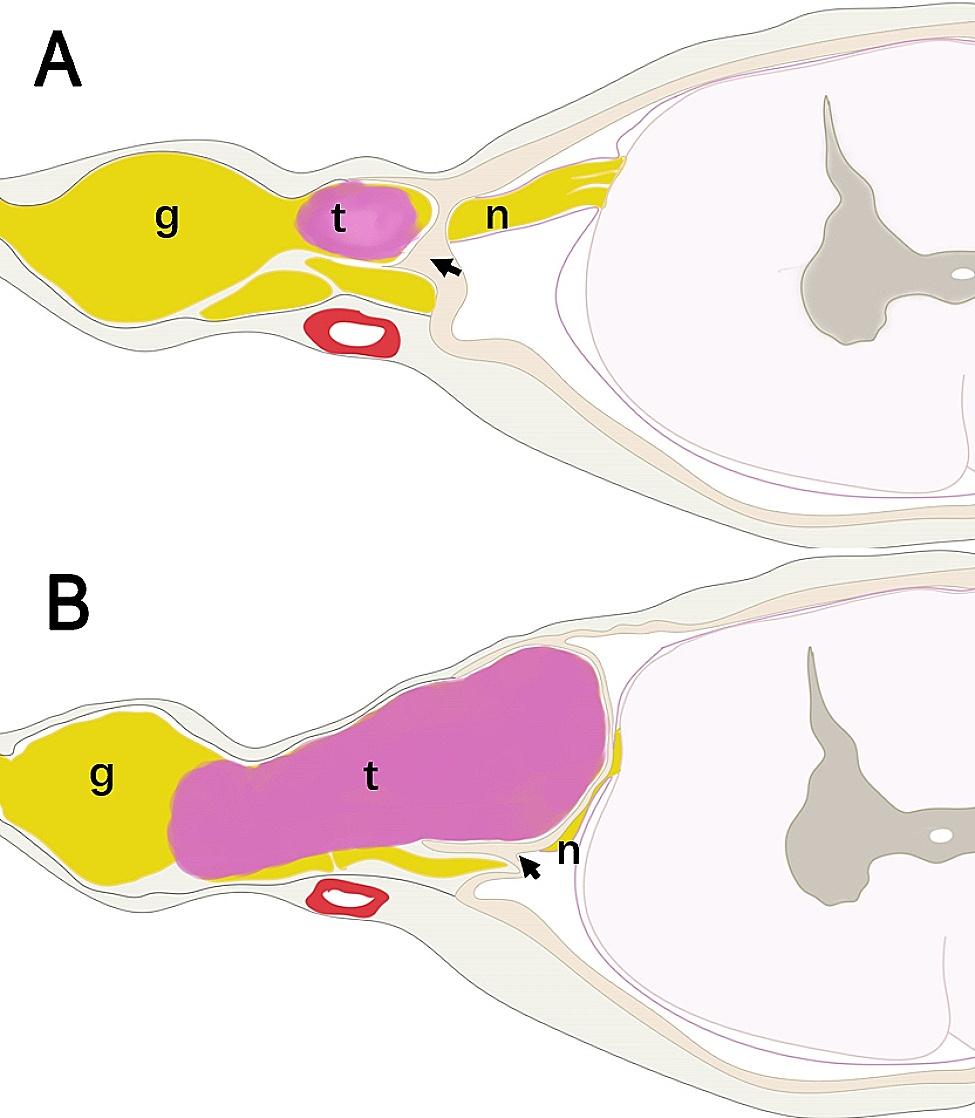
**A:** The dural sac forms a pocket-like structure (black arrow) receiving the nerve root, and we call it the dural ring. The extradural schwannoma that is located outside of the sheath of the dural ring. **B:** As the tumor grows, it grows in the intraspinal canal without invasion of the intradural portion. g: dorsal root ganglion, n: nerve root, t: tumor

Eden [5] proposed one of the most commonly used classification systems for dumbbell spinal schwannomas, which divides the schwannomas into four groups: (I) intra-/extradural, with no paravertebral component; (II) intra-/extradural with the paravertebral component; (III) extradural and paravertebral; and (IV) foraminal and paravertebral. The dumbbell-shaped tumor type with intradural and extradural components (Eden classification type I and II) is known to be associated with a higher incidence of complications (CSF leakage, pseudo meningocele, and wound infection for instance) and postoperative neurologic deterioration [4]. One possible reason is that the removal of this type of tumor results in dural defects and therefore necessitates a technically demanding dural repair [6]. Another reason could be that the removal of the extradural portion of the tumor together with the epineurium may inevitably injure the functioning nerve fibers that may reside in the epineurium [4]. About 3/4 of dumbbell tumors are completely restricted to the extradural space (Eden classification type III and IV), [7] although preoperative MRI in some cases suggests the presence of intradural/extradural tumors [8]. Sometimes it is challenging to preoperatively differentiate extradural dumbbell tumors from intradural/extradural tumors by imagining data [8]. The invagination of the dural ring (Fig. 1), composed of band-like tissue with a thin sheath around the nerve root, [9] is the anatomical feature that may cause confusion between the intradural/extradural tumor type (Eden classification type II) and the extradural dumbbell tumor (Eden classification type III) during surgery [8]. Many dumbbell tumors could be totally removed without durotomy, although these tumors seem to have an intradural/extradural mass [8]. Therefore, a new surgical strategy is needed when a completely extradural dumbbell tumor is confirmed during the procedure.

Here, we report on our experience with an alternative surgical strategy, referred to as the separate-dural-incision method, and extradural resection technique of dumbbell-shaped schwannoma with intraspinal and extraspinal components.

## Patients and methods

### Patients

This is a retrospective case series of patients treated at the Neurosurgery Department of the First Affiliated Hospital of the University of Science and Technology of China between January 2018 and June 2021 with the diagnosis of completely extradural dumbbell schwannoma. Inclusion criteria were the following: (1) patients received surgical treatment via one-stage posterior approach with hemi- or laminectomy and facetectomy for Eden type III spinal schwannomas; (2) histopathologic results compatible with spinal schwannoma; and (3) no previous treatment. The data were collected from the hospital charts: clinical status at admission, imaging results, histopathologic findings, surgical management, complications, and outcome. Preoperative assessments included CT and MRI and/or CT angiography. As a retrospective case series, only patients who met all inclusion criteria were analyzed. Owing to the retrospective nature of this study, the ethics committee of our hospital approved it to be exempt requiring informed consent from the patients.

### Surgical technique

**Fig. 2:**
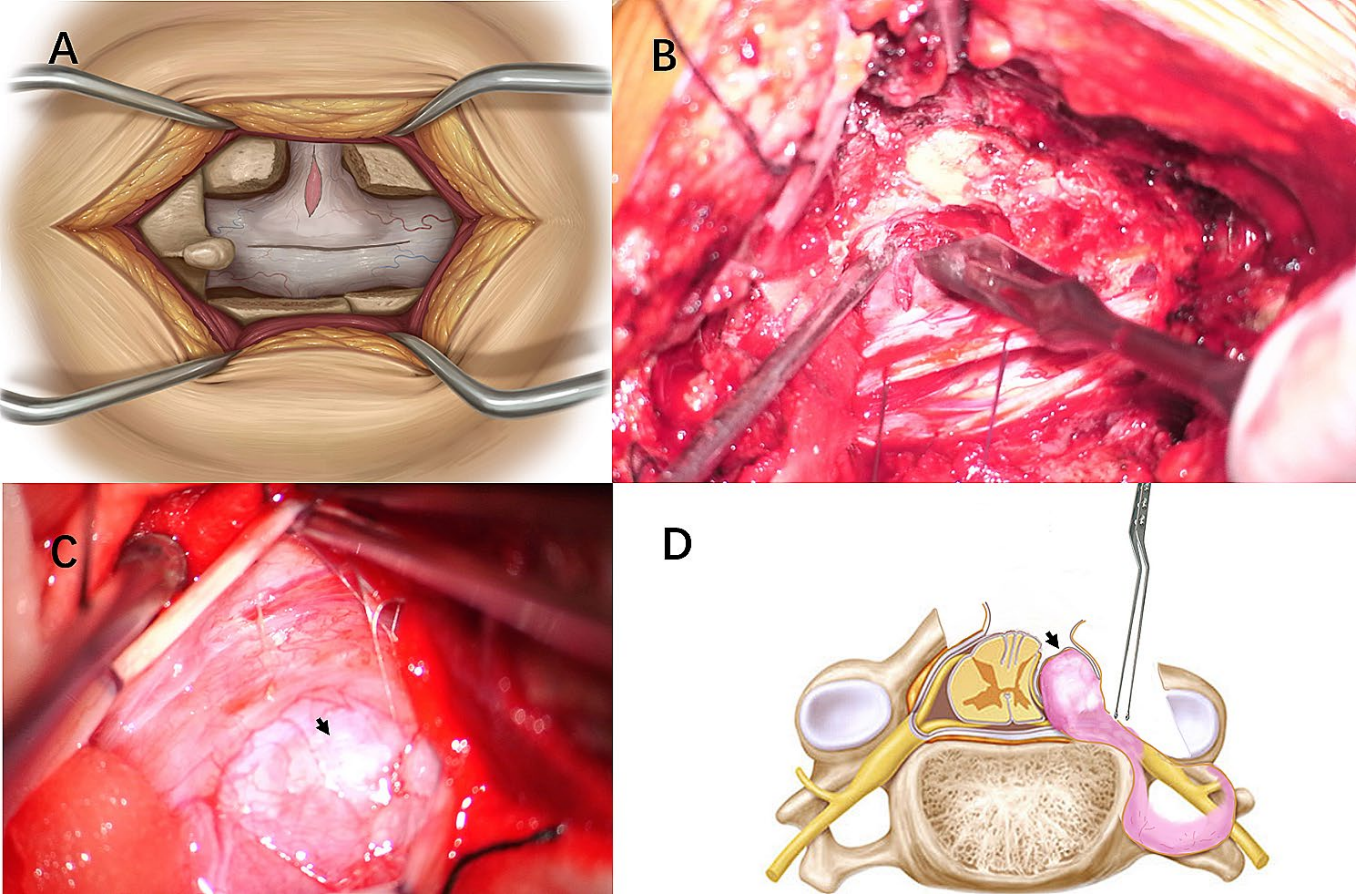
**A, B:** The dura mater is opened with 2 separate incisions. **C:** The intraoperative image of a patient showing the intraspinal portion of the tumor is covered with an attenuated dura (black arrow). **D:** Debulking of the extraspinal portion and sectioning of the tumor is performed through the incision along the nerve root. The picture also shows the dura covering the intraspinal portion of the tumor (black arrow)

**Fig. 3:**
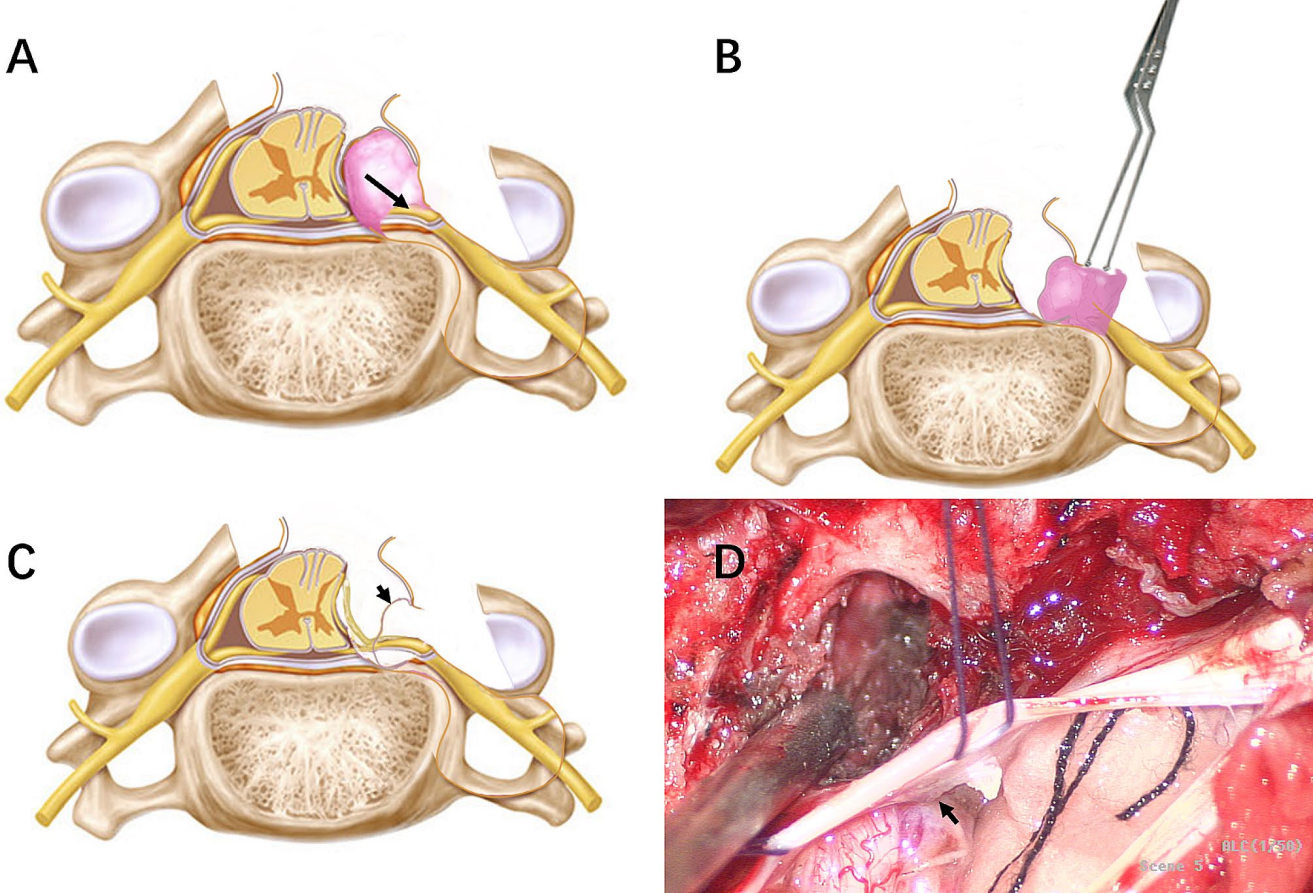
**A:** The intradural-like mass (the intraspinal component) is pushed out from the subdural space via the incision along the dural theca. The black arrow shows the direction of the push. **B:** The intraspinal component of the tumor was being removed through the incision along the nerve root. **C, D:** After the epidural removal of the intraspinal components, postresection inspection was performed via the dural incision along the dural theca to determine if there was a residual invaginated tumor. The dura (black arrow) covers on the intraspinal portion of the tumor is well preserved

After general anesthesia and intubation, the patient was placed in the prone position. Somatosensory evoked potential and motor-evoked potential were recorded. After a longitudinal midline skin incision, the fascia and paravertebral muscle were dissected subperiosteally from the spinous process and lamina. The muscle was dissected laterally to the lateral part of the affected side facet joint to expose the distal part of the tumor. The muscle traction was performed with the intervertebral foramen tumor as the center of the exposure. Hemilaminectomy or laminectomy, depending on the size of the intraspinal tumor component, was performed with a high-speed drill. Partial facetectomy was performed. The dura mater was opened with 2 separate incisions: one dural incision was made along the nerve root to allow visualization of the extraspinal portion of the tumor and tumor debulking. Another separate longitudinal dural incision was made along the dural theca to provide adequate visualization of the intraspinal portion of the tumor for confirmation of the relationship between the dura and tumors (Fig. 2, A and B). Surgery was conducted with the utmost to avoid connecting the separate dural incisions and to maintain the dura ring around the nerve root. The intraspinal portion of the tumor covered with an attenuated dura was confirmed (Fig. 2, C and D). The removal of the tumor was done in the following manner: For the external component of the vertebral foramen, it was resected piecemeal after retraction within the capsule through the incision along the nerve root (Fig. 2, D). Microsurgical dissection of the tumor was made just beneath the epineurium to preserve the viable nerve fibers. After removal of the extraspinal portion and sectioning of the tumor through the incision along the nerve root, the intraspinal component was pushed out from the subdural space to intervertebral foramen (Fig. 3, A), and removed via the incision along the nerve root (Fig. 3, B). The dura covered on the intraspinal portion of the tumor was well preserved (Fig. 3, C and D). Finally, the two separate dural incisions were closed with needle and thread suturing and the wound was well irrigated and closed in layers.

### Follow-Up and evaluation of results

**Fig. 4:**
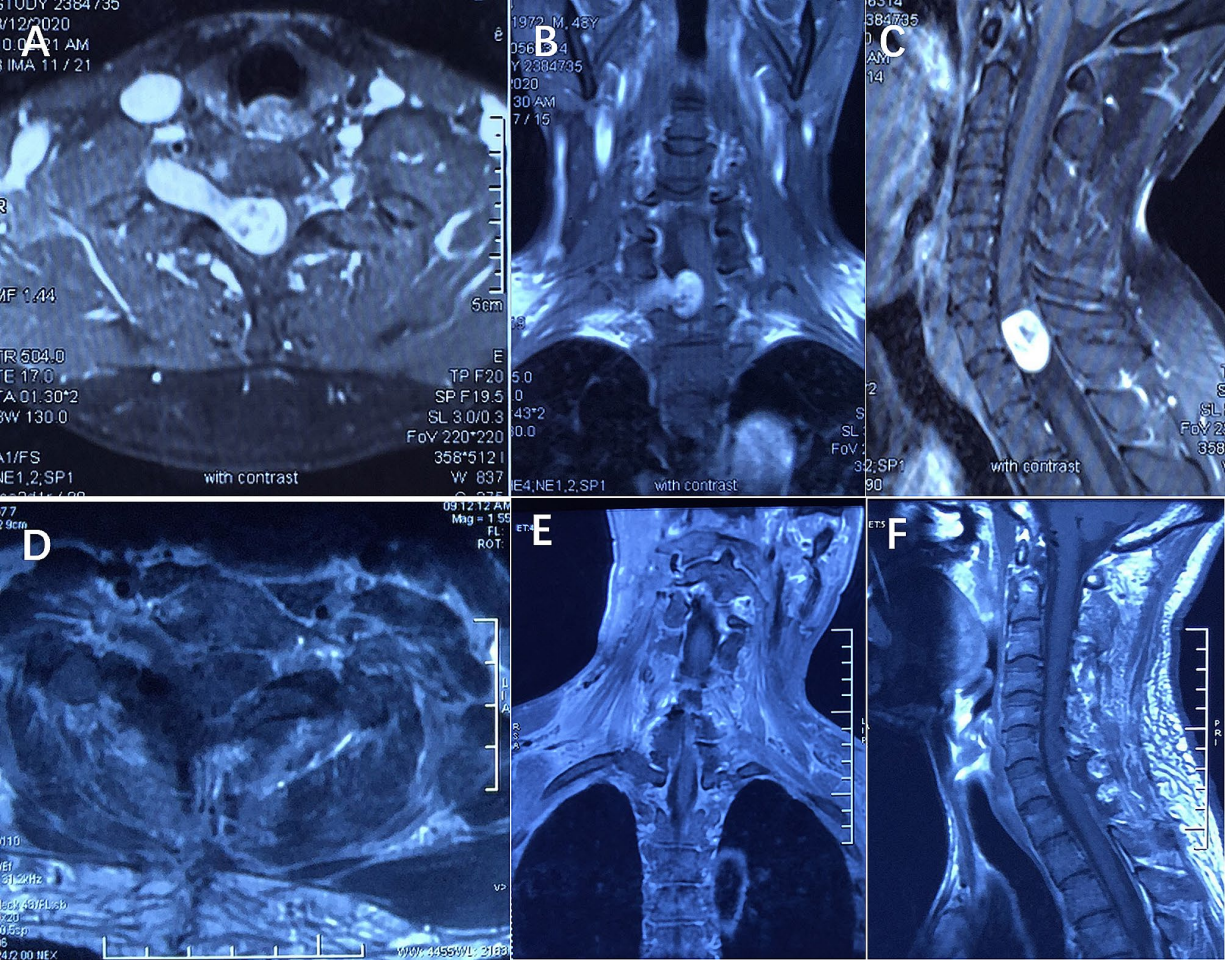
Preoperative and postoperative MR images of a 48-year-old male patient who complained of right upper extremity numbness and weakness. **A, B,** and **C:** The preoperative MRI showed that obvious intensified signals of the dumbbell tumor at the C7-T1 level, and it grew toward the right. **D, E,** and **F:** The postoperative enhanced MRI showed the total removal of the tumor

Enhanced MRI was performed one month after the surgery to determine if there was a residual tumor (Fig. 4). Gross total resection was defined as the absence of residual tumor on postoperative MRI.

## Results

### Clinical baseline data

Data for sex, age at surgery, disease duration, preoperative symptoms, and tumor location are shown in Table 1. There were 24 patients (16 male, 8 females; median age 56 years, range 5–75 years) enrolled in the study, with cervical and lumbar spines being the most frequent locations. Of the 24 patients, all of these tumors were symptomatic. The clinical symptoms were spinal pain (10 cases), motor weakness (8 cases), sensory disturbance of the lower extremities (12 cases), pain of the lower extremities (9 cases), gait disturbance (5 cases), and bladder and bowel dysfunction (2 cases). 7 cases were misdiagnosed as intradural/extradural tumor type (Eden classification type II) before surgery.

**Table 1:** Demographic data and surgical outcomes for 24 patients with dumbbell spinal schwannoma

Item		Value
Age (year)		Median 56 (range 5–75)
Sex		
Female		8 (33.3%)
Male		16 (66.7%)
Preoperative symptoms N (%)		
Spinal pain		10 (41.7%)
Motor weakness		8 (33.3%)
Sensory disturbance of the lower extremities		12 (50%)
Pain of the lower extremities		9 (37.5%)
Gait disturbance		5 (20.8%)
bladder and bowel dysfunction		2 (8.3%)
Duration of symptoms (month)		Median 6 (range 1–36)
Tumor Eden classification N (%)		
Type II		7 (29.1%)
Type III		17 (70.9%)
Tumor location N (%)		
Cervical		11(45.8%)
Thoracic		5(20.8%)
Lumbar		8(33.3%)
Operative time (min)		average 125 ± 23.8
Tumor resection		
Gross total resection		24(100%)
Subtotal resection		0
Postoperative sensory deficit		2(8.3%)
Postoperative motor deficit		0
Following up (month)		average 27.6 ± 12.1

### Surgery outcomes and follow-up

Dumbbell tumors were removed successfully by using the one-stage posterior approach in all cases. The average surgical time was 125 ± 23.8 min. Total resection was done in all the patients, among which 11 cases were treated with hemilaminectomy and 13 cases with laminectomy and facetectomy. The affected nerve roots were transected in 6 cases. Two cases had numbness and no case exhibited motor deficit. There was no cerebrospinal fluid leakage or central nervous system infection. The average follow-up was 27.6 ± 12.1 months, with a range of 6–46 months, none postoperative spinal instability occurred. The MRI showed that there was no tumor reoccurrence.

### Case examples

#### Case 1

**Fig. 5:**
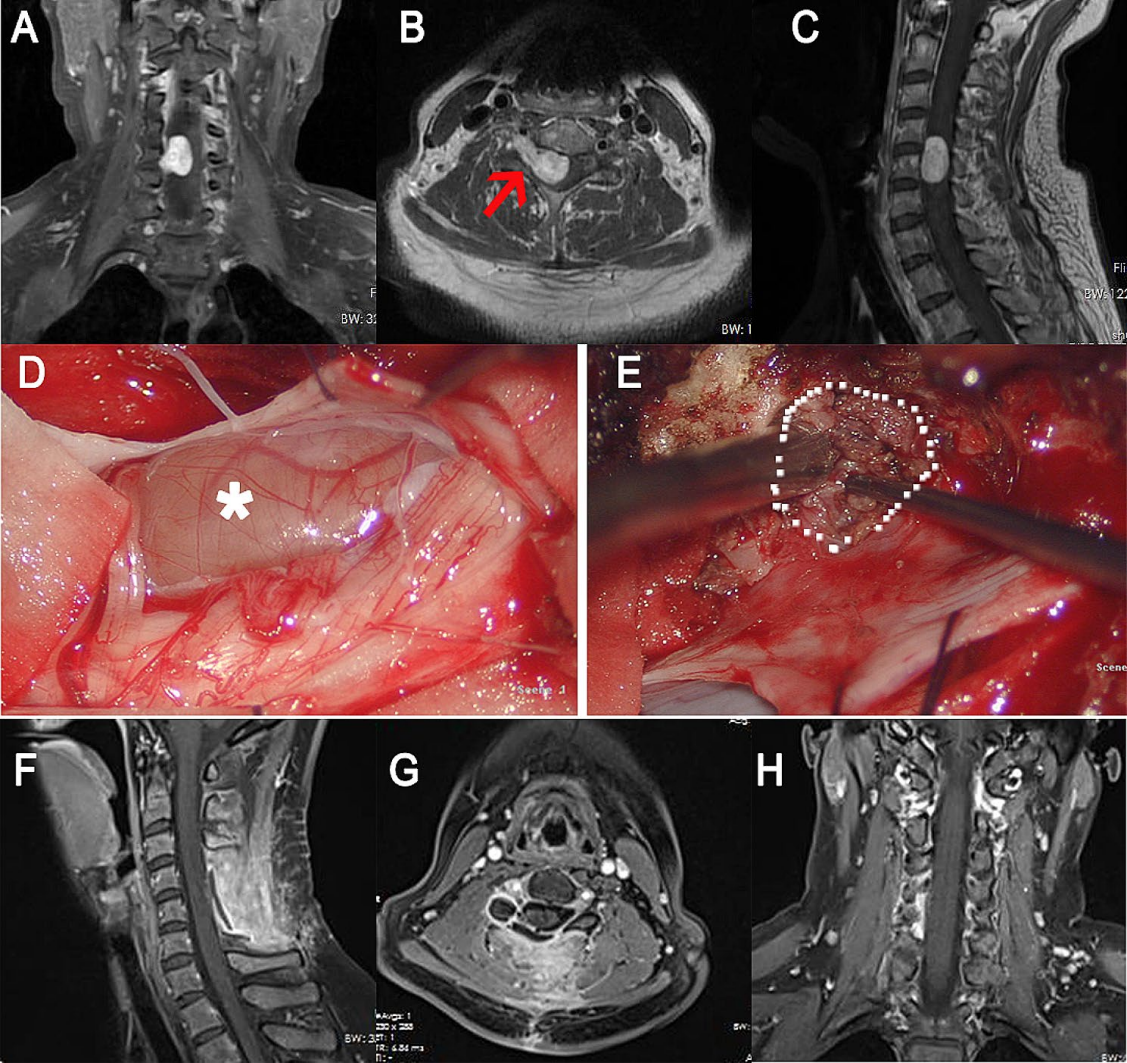
A 40-year-old woman was diagnosed with spinal schwannoma. **A, B**, and **C**: preoperative MR images. The tumor (red arrow) was located at the C4-5 level. **D** and **E**: images during the operation. The white star indicated the intraspinal portion and the white dot line showed another incision to expose the extraspinal portion. **F**, **G**, and **H**: MRI performed one month after the operation

(Fig. 5): A 40-year-old woman complained of continuous numbness in her right arm for over 1 year, combined with a feeling of pain recently. On physical examination, she was found to have hypoesthesia to light touch sensations and mild weakness (4/5) of the right upper limb. Preoperative MR images demonstrated an intradural mass at the C4-5 level, extending out to the right C5-6 neural foramen. The separate-dural-incision method was utilized in the surgery and total resection was achieved. One month after the operation, there was no tumor residue in the MRI, and she had no numbness or pain. In two years of follow-up, no issue existed.

#### Case 2

**Fig. 6:**
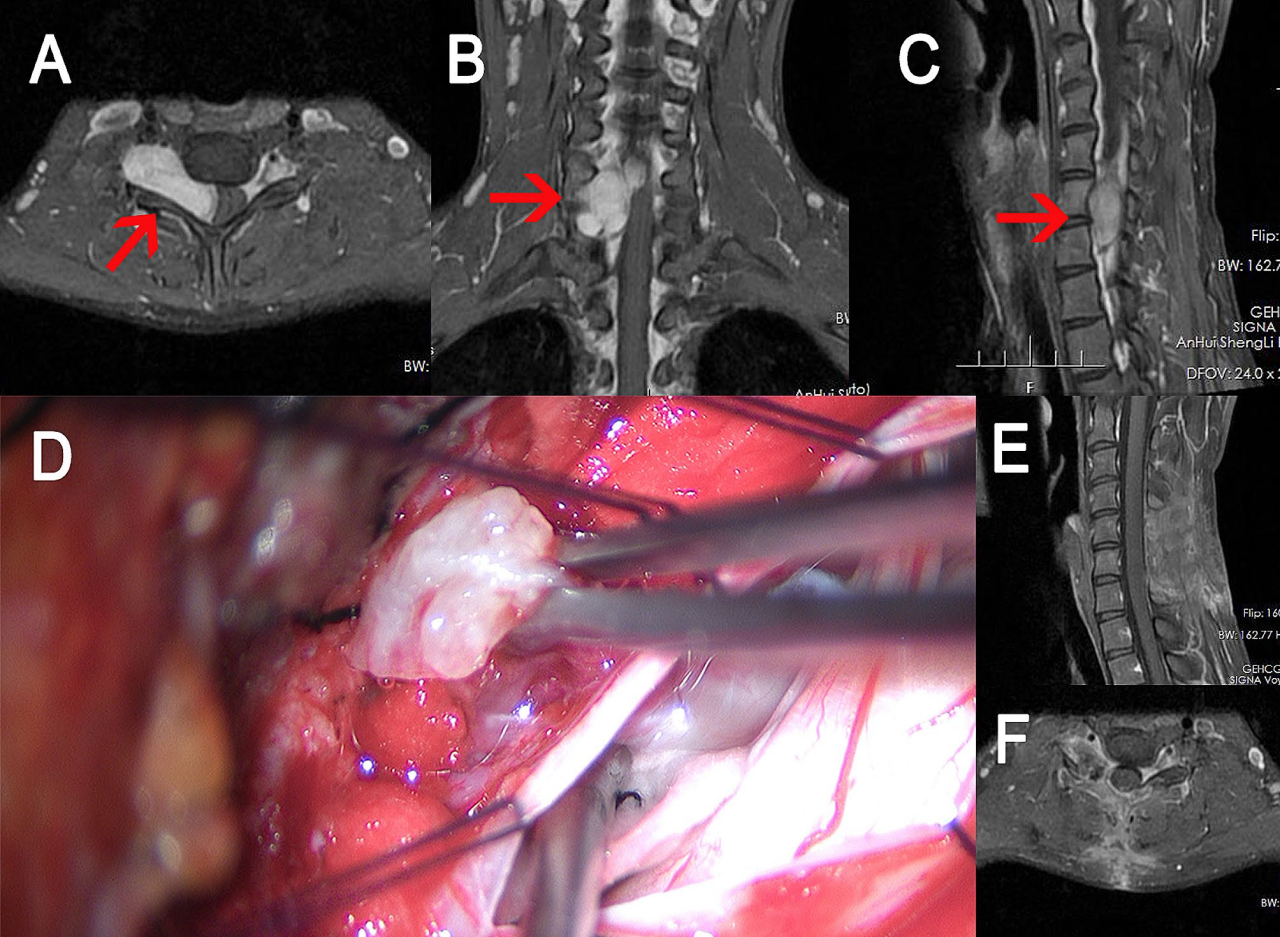
The data of case 2. **A, B**, and **C**: preoperative MR images. The tumor (red arrow) was located at the C6-7 level. **D**: images during the operation. Pulling the tumor through one incision combined with pushing through another incision. E and F: MRI performed two months after the operation

(Fig. 6): A 17-year-old boy suffered mild numbness in his right arm for one year, with left arm weakness for one month. Neurological examination revealed muscle strength of the left upper limb was grade 4. A Cervical spine MRI showed an extramedullary enhanced mass in the C6-7 level and right paravertebral region, with a typical dumbbell shape. He was discharged one week after the operation with normal muscle strength. There was no tumor recurrence or other issues during the one-year follow-up.

An operative video of the whole procedure was illustrated in this link: 

https://youtube.com/playlist?list=PLcXX2tgphbhllPic1k8Lh0kYqHBQTXXez&si=QgA_LW0bdoQF6Ukm

## Discussion

Most of the dumbbell tumors are completely restricted to the extradural space, [7] although preoperative MRI in some cases suggests the presence of intradural/extradural tumors [8]. The extradural tumor located outside of the sheath of the dural ring can grow in the intraspinal canal without invasion of the intradural portion, which leads to confusion with an intradural/extradural tumor (Eden classification type II) during operations for extradural dumbbell tumors (Eden classification type III) [8]. The dumbbell-shaped tumor type with intradural and extradural components is known to be associated with a higher incidence of complications and postoperative neurologic deterioration [4]. Thus, traditional surgical strategy, when ignore the surgical importance of the invagination of the dural ring in extradural dumbbell tumors, for extradural dumbbell-shaped tumor might be associated with a higher incidence of complications and postoperative neurologic deterioration. In this article, we retrospectively reported a case series of extradural dumbbell tumors (Eden classification type III and type II) resected with an alternative surgical strategy, and found all of the patients had satisfactory tumor resection with a low incidence of postoperative complications.

Good surgical results have been recently reported demonstrating feasibility, safety, and effectiveness of minimally invasive techniques in the treatment of extradural schwannomas [10, 11]. Extradural dumbbell tumors could be removed without durotomy, and the risk of the cerebrospinal fluid leak was low [10, 11]. However, sometimes it is challenging to preoperatively differentiate extradural dumbbell tumors from intradural/extradural tumors by imagining data [8]. In our case series, 7 cases of extradural dumbbell spinal schwannoma were preoperatively misdiagnosed as intradural/extradural tumors. Surgery for this type of tumor often utilizes a T-shaped dural incision, which is made parallel to the spinal canal and nerve root to provide good visualization of the tumor [12, 13], while a dural defect is often created in the area where the incisions cross [13]. Subdural removal of the intraspinal component requires additional time for dural repair because of the large size of the defect and irregularity of its margin [8]. The dural defects and needs of a technically demanding dural repair after tumor resection are associated with a higher incidence of complications such as CSF leakage, pseudo meningocele, and wound infection [6]. The separate-dural-incision method made in our case series provided a surgery field to confirm the relation between the dura and tumors. After the epidural removal of the intraspinal components, postresection inspection was performed via the dural incision along the dural theca to determine if there was a residual invaginated tumor. The incision along the nerve root is the main one, which aims to expose the extradural tumor and provide the surgery field for resection. Thus, for some experienced surgeons, the longitudinal dural incision may not needed when removing the extradural tumor portion and extracting the intradural component totally, especially in some typical Eden Type III cases.

Indeed, one incision without longitudinal dural incision achieved total resection is more minimally invasive theoretically. Actually, we had attempted this method in a few cases but all finally gave up. Summarizing our failure, we found that this method requires many skills to accomplish. Some small vessels and nerve fibers may tightly attach to the tumor capsule, and pulling out the tumor increases bleeding and neuro injury possibility. Without a longitudinal dural incision, the direct vision of subdural space is limited, and the confirmation of no tumor residence is hard. Besides, we applied two incisions in all cases of this study based on some other considerations. First, we mainly relied on pushing the intradural tumor portion out through the dural ring rather than pulling it out. The volume of the intradural portion was larger than the dural ring size due to its dumbbell shape, solely pulling out this part may be challenged. Combined with the push strength makes it easier. Then, the manipulation in subdural space aimed to dissociate tumor attachment, almost using a blunt stripper rather than sharp instruments. It increased invasion to normal tissue barely but decreased injury possibility in extraction. Furthermore, the suture of a longitude incision was easy and reliable, without adding issues like CSF leakage. The concept of “minimally invasive” should not be limited to the numbers or size of incisions, more concerns should be paid to avoiding injury of the key tissues like nerves and vessels and decreasing the likelihood. Thus, we preferred the two-incision method and also supported other skillful surgeons who are capable of controlling the injury risk to attempt the one-incision method. Compared to traditional T shape incision, the separate-dural-incision method preserved the integrity of the dura mater as much as possible, and enabled the surgeon to reduce the likelihood of CSF leakage with the use of a simpler and more reliable dural closure method for linear incisions. Kiyoshi ito [13] reported the same dural incision and found it was preferable to the conventional T-shaped dural incision method because no dural defects occurred after the intradural procedure and meticulous dural closure was achieved.

Gross total resection with preservation of neurological functions is the best treatment to relieve patients’ complaints and to reduce the recurrence rate of spinal schwannoma [4, 14]. Whether sacrificing the parent nerve root is necessary to achieve a gross total resection and to reduce the risk of tumor recurrence remains debatable, as does the risk of a postoperative permanent deficit [15, 16]. Though debated, cutting nerve root is a relatively common choice during schwannomas surgery [14]. Amputating critical parent nerve roots during the dumbbell tumor resections seems to result in a low incidence of postoperative motor deficits [17]. However, schwannomas, despite being rare, originating from motor roots are reported almost in every surgical series, and in some cases, the severe postoperative motor deficit was observed when the nerve was cut [4, 16]. Debate about this point is ongoing, as today’s goal of surgical treatment is not anymore considered just tumor removal, but also preserving the patient’s quality of life [14]. Motor deterioration was related to preoperative motor weakness, preoperative gait disturbance, dumbbell Eden type II tumor, subtotal resection, and operative time; whereas sensory deterioration was related to preoperative gait disturbance and subtotal resection [18]. Subdural resection of the intraspinal portion of extradural dumbbell schwannoma might increase the risk of nerve root and spinal cord injury, and lead to blood accumulation in the subdural/ subarachnoid space. Jose Poblete [10] reported minimally invasive surgical technique for 15 cases of giant extradural dumbbell spinal schwannoma. Gonçalves [11] introduced a case of resection of a completely extradural lumbar schwannoma through a minimally invasive approach using an expandable trans muscular tubular retractor. Total gross resection was accomplished in all patients and durotomy or spine instrumentation was not necessary. However, extradural removal of the intraspinal mass, if not under a direct vision, might increase the risk of injury to the spinal cord, which is vulnerable after a long-term compression of the tumor. Our extradural removal techniques of dumbbell tumors, to push the intradural-liked mass out from the subdural space, using a blunt tripper to isolate the tumor with spinal cord and parent nerve root rather than direct resection, was associated with a lower incidence of postoperative neurologic deterioration.

## Conclusion

Based on a limited number of observations, we conclude that our technique was feasible and effective for the treatment of extradural dumbbell spinal schwannomas.

### Limitations

This study’s limitations include a small sample size and a lack of concomitant randomized controlled cases performed by the same experienced surgeon who performed traditional operations.

## Data Availability

The data that support the findings of this study are available from the corresponding author upon reasonable request.
